# Inferring within‐herd transmission parameters for African swine fever virus using mortality data from outbreaks in the Russian Federation

**DOI:** 10.1111/tbed.12748

**Published:** 2017-11-09

**Authors:** C. Guinat, T. Porphyre, A. Gogin, L. Dixon, D. U. Pfeiffer, S. Gubbins

**Affiliations:** ^1^ Veterinary Epidemiology, Economics and Public Health Group Royal Veterinary College Hatfield Hertfordshire UK; ^2^ The Pirbright Institute Pirbright Surrey UK; ^3^ The Roslin Institute University of Edinburgh Roslin Midlothian UK; ^4^ European Food Safety Authority Parma Italy; ^5^ Federal Research Center for Virology and Microbiology Pokrov Russia; ^6^ College of Veterinary Medicine & Life Sciences City University of Hong Kong Kowloon Hong Kong; ^7^ Present address: École Nationale Vétérinaire de Toulouse Toulouse France

**Keywords:** approximate Bayesian computation, disease control, epidemiology, modelling, mortality data, pigs

## Abstract

Mortality data are routinely collected for many livestock and poultry species, and they are often used for epidemiological purposes, including estimating transmission parameters. In this study, we infer transmission rates for African swine fever virus (ASFV), an important transboundary disease of swine, using mortality data collected from nine pig herds in the Russian Federation with confirmed outbreaks of ASFV. Parameters in a stochastic model for the transmission of ASFV within a herd were estimated using approximate Bayesian computation. Estimates for the basic reproduction number varied amongst herds, ranging from 4.4 to 17.3. This was primarily a consequence of differences in transmission rate (range: 0.7–2.2), but also differences in the mean infectious period (range: 4.5–8.3 days). We also found differences amongst herds in the mean latent period (range: 5.8–9.7 days). Furthermore, our results suggest that ASFV could be circulating in a herd for several weeks before a substantial increase in mortality is observed in a herd, limiting the usefulness of mortality data as a means of early detection of an outbreak. However, our results also show that mortality data are a potential source of data from which to infer transmission parameters, at least for diseases which cause high mortality.

## 1 INTRODUCTION

Mortality data are routinely collected for livestock and poultry species, either as part of herd management practices (e.g., in pigs and poultry) or as part of mandatory animal movement reporting or monitoring of animals found dead on farm (e.g., in cattle). These data have often been used for epidemiological purposes, for example, as part of syndromic surveillance (Alba et al., 2015; Tapprest et al., 2016; Torres et al., 2015), for detecting outbreaks of disease (Backer, Brouwer, van Schaik, & van Roermund, 2011; Bos et al., 2007) or to assess the impact of an epidemic (Perrin et al., 2010).

For diseases which cause very high mortality, such as highly pathogenic avian influenza, mortality rates provide a proxy for the incidence of newly infected animals. In this case, back‐calculation methods can be used to estimate transmission parameters from mortality data (Bos et al., 2009; Tiensin et al., 2007). Typically, these back‐calculation approaches assume that the latent and infectious periods are known and fixed to facilitate implementation of the methods. This raises the question of whether it is possible to estimate transmission parameters from mortality data without needing to make such assumptions about the latent and infectious periods. In this study, we explore this question using African swine fever as a case study.

African swine fever (ASF) is one of the most important infectious diseases of swine (Costard, Mur, Lubroth, Sánchez‐Vizcaíno, & Pfeiffer, 2013; Sánchez‐Vizcaíno, Mur, Gomez‐Villamandos, & Carrasco, 2015). It is caused by African swine fever virus (ASFV) and many strains result in the death of almost 100% of infected pigs (Blome, Gabriel, & Beer, 2013; Guinat, Gogin, et al., 2016). African swine fever virus is endemic to sub‐Saharan Africa and, apart from in Sardinia where it is also endemic, most previous incursions of the virus into Europe or the Americas have been successfully controlled (Costard et al., 2009). Following an incursion of ASFV to Georgia in 2007 (Rowlands et al., 2008), however, the virus subsequently spread throughout the Caucasus, the Russian Federation (RF), the Baltic States and into Eastern Europe (EFSA Panel on Animal Health and Welfare, 2014; Guinat, Gogin, et al., 2016).

Here, we fit a stochastic model for the within‐herd transmission of ASFV to mortality data for nine pig herds in the RF affected by confirmed ASFV outbreaks. Parameters were estimated using approximate Bayesian computation (McKinley, Cook, & Deardon, 2009; Toni, Welch, Strelowa, Ipsen, & Stumpf, 2009), paying particular attention to the sensitivity of the estimates to the prior assumptions made. This allows us to address the question of what can be inferred about ASFV transmission using mortality data.

## 2 MATERIALS AND METHODS

### 2.1 Mortality data

**Figure 1:**
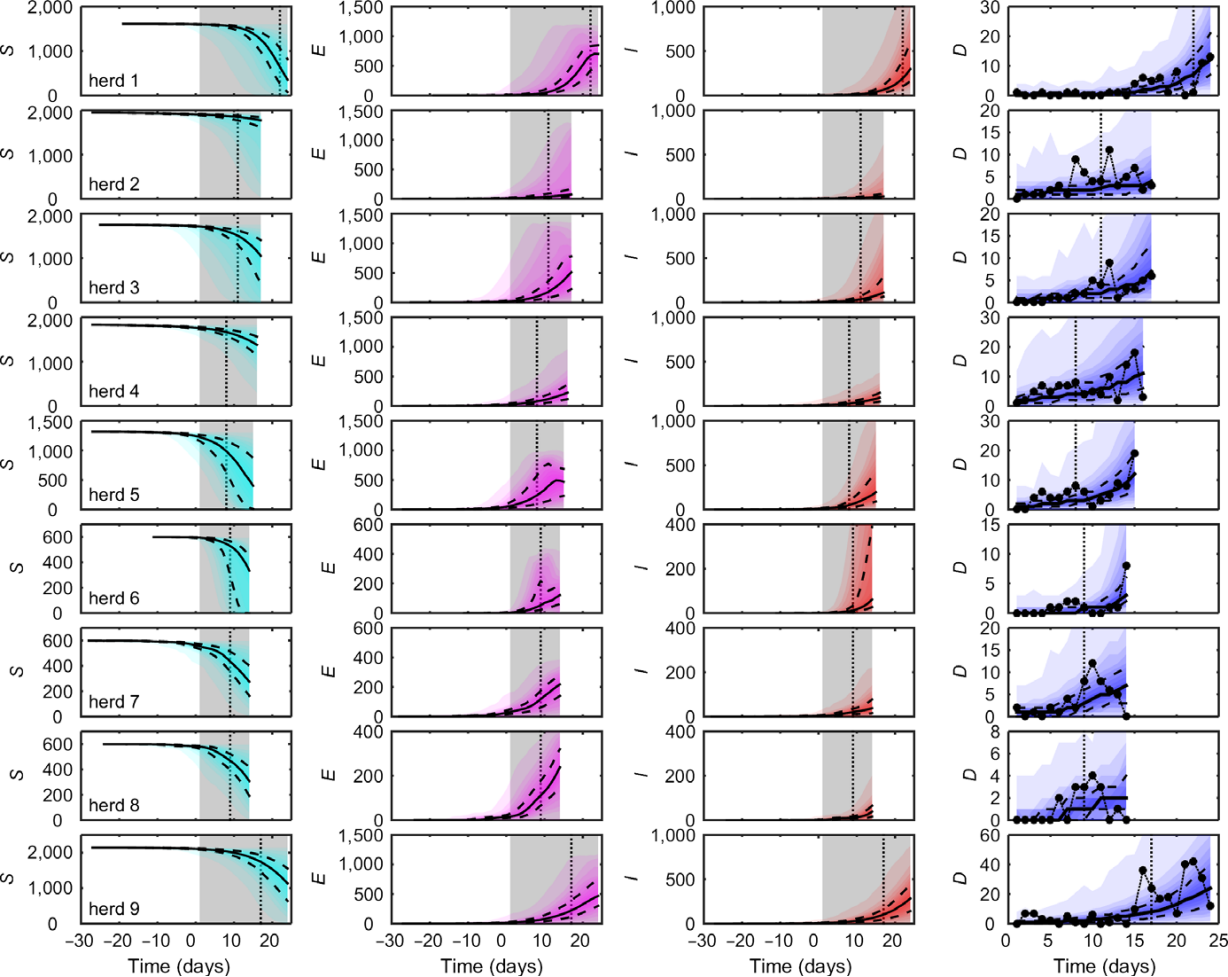
Dynamics of African swine fever virus (ASFV) in nine pig herds in the Russian Federation. Each column shows the predicted number of susceptible (S, cyan), exposed (E, magenta), infectious (I, red) and dead pigs (D, blue), respectively, as well as the observed number of dead pigs. Predicted dynamics are shown as the median (solid black line), 25th and 75th percentiles (dashed black lines) and five percentile bands (up to the 5th and 95th percentiles; shading). Observed daily mortalities are shown as black circles (last column). The grey shaded area indicates the observation period for the mortality data, and the black dotted line indicates the day on which ASFV was confirmed in the herd. Results are based on 1,000 replicates of the model sampling from the joint posterior distribution assuming informative priors for all parameters

Incidence and testing data for ASFV in the RF are routinely collected by the Federal Research Center for Virology and Microbiology (FRCVM). Data on pig mortality were obtained for nine pig herds in which ASFV was detected through routine surveillance between 2010 and 2014 (Table S1; see also Figure 1). These herds were selected based on the availability and quality of the data collected on pig mortality. All nine were industrial pig herds, implementing intensive indoor production systems with stringent biosecurity measures. Herd sizes ranged from 600 to 2,145 fattening pigs, with a median size of 1,614. They were located in south‐west (herds 2, 3, 4, 5, 6, 7 and 8) and north‐west (herds 1 and 9) regions of the RF. The outbreaks took place in summer (herds 1, 2, 3, 4 and 5) or winter (herds 6, 7, 8 and 9). During the observation period, an increase in the numbers of dead pigs and clinical signs suggestive of ASF (depression, loss of appetite, redness of the skin and fever) was reported by farmers. African swine fever virus infection was confirmed in all nine herds by virus isolation from randomly collected blood and tissue samples taken from dead pigs.

### 2.2 Modelling approach

Although it is maintained in a sylvatic warthog–tick cycle in sub‐Saharan Africa, once introduced to domestic pigs, ASFV is transmitted through direct contact and fomites (Costard et al., 2013). Accordingly, the within‐herd dynamics of ASFV were modelled using a stochastic *SEIR* epidemic model (Keeling & Rohani, 2008). In the model, the pig population is divided into three classes: susceptible (i.e., uninfected), S, exposed (i.e., infected but not yet infectious), E, and infectious, I. There is little evidence that pigs recover following infection with ASFV strains circulating in the RF (Guinat, Gogin, et al., 2016) and, consequently, all pigs were assumed to die at the end of their infectious period (so the removed class, *R*, is not needed for the model).

The force of infection is given by,

(1) $$\lambda \left(t\right)=\beta \frac{I(t)}{N(t)},$$

where β is the transmission rate, *I*(*t*) is the number of infectious pigs and *N*(*t*) is the total number of pigs at time *t*. This formulation assumes homogeneous mixing (i.e., individuals uniformly and randomly contact each other) and frequency‐dependent transmission (i.e., the number of contacts is independent of the population size) (Keeling & Rohani, 2008). The durations of the latent and infectious periods were assumed to follow gamma distributions with means μ_E_ and μ_I_ and shape parameters *k*_E_ and *k*_I_, respectively. This was incorporated in the model by subdividing the latent and infectious classes into *k*_E_ and *k*_I_ stages each of mean duration μ_E_/*k*_E_ and μ_I_/*k*_I_, respectively (Anderson & Watson, 1980). Natural mortality (i.e., not associated with ASF) was included at a *per capita* rate *r*_*M*_ for all classes in the model.

Assuming that natural mortality occurs at a low rate, the basic reproduction number (*R*_0_), defined as “the average number of secondary cases caused by an average primary case in an entirely susceptible population” (Keeling & Rohani, 2008), is approximated by,

(2) $$R_{0}=\beta \mu_{I}$$

Population sizes in the model take integer values, while transitions between compartments are stochastic processes (Table S2). The number of transitions of each type during a small time interval δ*t* was drawn from a binomial distribution with population size *n* and transition probability *q* (the appropriate *per capita* rate multiplied by δ*t*); (Table S2). However, binomial random variables are computationally expensive to simulate and an approximating distribution was used wherever possible. If: (i) *nq*(1 − *q*) > 25; (ii) *nq*(1 − *q*) > 5 and 0.1 < *q *<* *0.9; or (iii) min(*nq*,* n*(1 − *q*)) > 10, an approximating normal variate with mean *nq* and variance *nq*(1 − *q*) was used, while if *q *<* *0.1 and *nq *< 10, an approximating Poisson variate with mean *nq* was used (Forbes, Evans, Hastings, & Peacock, 2011).

### 2.3 Approximate bayesian computation

The transmission rate (β), latent and infectious period parameters (μ_E_, *k*_E_, μ_I_, *k*_I_) and natural mortality rate (*r*_*M*_) were estimated for each herd independently. In addition, the time at which ASFV was introduced to each herd (*t*_intro_) is unknown and was also estimated. Accordingly, seven parameters were estimated by fitting the model to the mortality data for each of the nine pig herds.

Parameters were estimated using approximate Bayesian computation sequential Monte Carlo (ABC‐SMC) methods (McKinley et al., 2009; Toni et al., 2009). Briefly, ABC‐SMC combines a particle filtering method with summary statistics and is ideal for stochastic models when the likelihood is difficult to define. Initial parameter sets (or particles) are sampled from a multivariate prior distribution, and then for subsequent SMC rounds with a perturbation kernel. After each model replicate (where a replicate involves picking one set of parameters and one stochastic simulation), the model and data are compared using a goodness‐of‐fit metric (defined below for the ASFV model). A parameter set is accepted if the distance between the model and data is less than a threshold defined by the previous SMC round. The accepted parameter sets from the final SMC round approximate the posterior distribution. The algorithm is described in Appendix S1, including details of the number of SMC rounds and the thresholds used.

The daily mortality data for each herd was used to define the goodness‐of‐fit metric. Specifically, the residual sum of squares for the daily mortalities was used as the goodness‐of‐fit metric for each herd, that is,

(3) $$D=\sum_{t=t_{1}}^{t_{2}}{\left(M_{\mathrm{sim}}\left(t\right)-M_{\mathrm{obs}}\left(t\right)\right)}^{2},$$

where *M*_sim_(*t*) and *M*_obs_(*t*) denote the simulated and observed number of dead pigs in the herd on day *t*, respectively, with the observation period for the herd running from day *t*_1_ to day *t*_2_.

Informative priors were constructed based on data for ASFV strains circulating in the RF (Guinat, Gubbins, et al., 2016; Gulenkin, Korennoy, Karaulov, & Dudnikov, 2011; Hu, Gonzales, & Gubbins, 2017) (Table S3). Gamma distributions were used for the transmission rate, β (mean 2, shape 2), the mean duration of the latent period, μ_E_ (mean 6.25, shape 10), the shape parameter for the latent period, *k*_E_ (mean 19.39, shape 5), the mean duration of the infectious period, μ_I_ (mean 9.12, shape 10), and the shape parameter for the infectious period *k*_I_ (mean 22.20, shape 5). An exponential prior was used for the natural mortality rate *r*_*M*_ (mean 0.0002, calculated from daily mortality data obtained from 34 ASFV‐free pig herds in the areas surrounding the outbreak herds). Finally, a uniform prior was used for the time at which ASFV was introduced to a herd, with a range from 30 days before the first observation to the time at which ASFV was confirmed in the herd (see Table S1). All priors were assumed to be independent of one another.

A second, non‐informative uniform prior with a wide range was also constructed for each parameter (Table S3). To explore the sensitivity of parameter inferences to prior assumptions, parameters were estimated for each herd using a further five combinations of priors (i.e., in addition to informative priors for all parameters): informative priors for all parameters, except the transmission rate; informative priors for all parameters, except the transmission and mortality rates; informative prior for the transmission rate only; informative prior for the mortality rate only; and non‐informative priors for all parameters.

## 3 RESULTS

### 3.1 Dynamics of African swine fever virus

**Figure 2:**
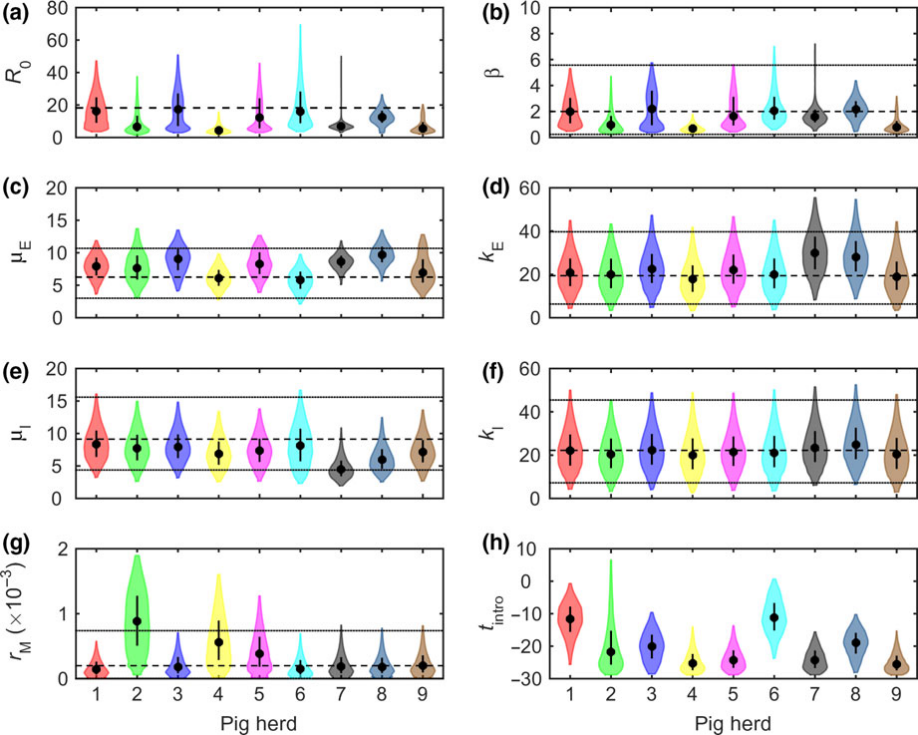
Transmission parameters for African swine fever virus (ASFV) inferred using mortality data for nine pig herds in the Russian Federation with confirmed outbreaks of ASFV. Plots show the marginal posterior distributions for the parameters for each herd: (a) basic reproduction number (*R*_0_); (b) transmission rate (β); (c) mean latent period (μ_E_); (d) shape parameter for latent period (*k*_E_); (e) mean infectious period (μ_I_); (f) shape parameter for infectious period (*k*_I_); (g) natural mortality rate (*r*_*M*_); and (h) time of introduction (*t*_intro_). The violin plots show the posterior density (shape), median (circle) and interquartile range (line) for the parameter (column) for the parameter. The dashed and dotted lines indicate the mean and 2.5th and 97.5th percentiles, respectively, for the informative prior distribution used for the parameter

The observed and predicted number of dead pigs and the predicted dynamics of ASFV are shown in Figure 1 for nine herds in the RF with confirmed outbreaks. The model captures the overall trend in mortality in each herd, and the observed daily mortalities lie within the 95% posterior prediction intervals in all instances (Figure 1). The predicted dynamics of ASFV underlying the observed mortality suggest that the number of latently infected pigs either had yet to reach or had just reached its peak at the time of culling in all herds (Figure 1). Similarly, the number of infectious pigs was predicted to be still increasing when the herd was culled (Figure 1). In addition, the number of dead pigs followed the number of infectious pigs with a delay of around one infectious period. Finally, the model predicts that ASFV was introduced to all these herds several weeks before the start of the observation period for the mortality data and, hence, several weeks before the day on which ASFV was confirmed in each of the herds (Figure 2h).

### 3.2 Parameter estimates

The basic *R*_0_ varied amongst the nine ASFV outbreaks, ranging from 4.4 to 17.3, and was significantly above one for all herds (Table 1; Figure 2a). This between‐herd variation in *R*_0_ is primarily a result of differences amongst herds in the transmission rate (β); (range: 0.7–2.2) but is also partly a consequence of differences in the mean duration of the infectious period (μ_I_); (range: 4.5–8.3 days); (Table 1; Figure 2b,e). The mean duration of the latent period (μ_E_) also differed amongst herds (range: 5.8–9.7 days); (Table 1; Figure 2c), though this is of limited epidemiological importance. The shape parameters for the latent and infectious periods (*k*_E_ and *k*_I_) did not differ greatly amongst herds (Table 1; Figure 2d,f). Finally, the natural mortality rate (*r*_*M*_) was similar in six of the herds, but somewhat higher in the remaining three herds (Figure 2g).

**Table 1:** Transmission parameters for African swine fever virus estimated using mortality data from outbreaks in nine pig herds in the Russian Federation

Parameter	Herd								
	1	2	3	4	5	6	7	8	9
Basic reproduction number (*R*_0_)									
Median^a^	16.2	6.7	17.3	4.4	12.2	15.9	6.9	12.6	5.6
95% CI^b^	(4.3, 41.7)	(1.9, 32.3)	(3.5, 45.5)	(2.0, 13.4)	(3.5, 40.5)	(4.5, 58.7)	(3.7, 41.3)	(3.7, 23.7)	(2.4, 18.4)
Transmission rate (β)									
Median^a^	2.0	1.0	2.2	0.7	1.6	2.1	1.6	2.2	0.8
95% CI^b^	(0.5, 4.8)	(0.3, 4.0)	(0.5, 5.3)	(0.3, 1.6)	(0.5, 5.1)	(0.7, 6.1)	(0.7, 6.4)	(0.6, 4.0)	(0.4, 2.8)
Mean latent period (μ_E_) (days)									
Median^a^	7.9	7.7	9.0	6.1	8.3	5.8	8.6	9.7	7.0
95% CI^b^	(4.3, 11.3)	(3.6, 13.0)	(4.7. 12.8)	(3.2, 9.3)	(4.4, 12.2)	(2.6, 9.1)	(5.9, 11.2)	(6.5, 12.9)	(3.5, 12.2)
Latent period shape (*k*_E_)									
Median^a^	20.8	19.9	22.5	17.8	22.0	19.9	29.8	28.1	18.9
95% CI^b^	(6.2, 40.9)	(5.5, 39.7)	(6.7, 43.0)	(4.7, 37.6)	(6.8, 42.6)	(5.5, 41.2)	(11.2, 51.5)	(11.3, 50.0)	(4.8, 40.5)
Mean infectious period (μ_I_) (days)									
Median^a^	8.3	7.7	7.9	6.9	7.4	8.2	4.5	6.0	7.2
95% CI^b^	(3.9, 14.8)	(3.3, 13.8)	(3.9, 13.6)	(3.0, 12.3)	(3.2, 12.8)	(2.9, 15.3)	(2.2, 9.5)	(3.0, 11.3)	(3.3, 12.5)
Infectious period shape (*k*_I_)									
Median^a^	22.0	20.3	22.2	20.0	21.5	21.1	23.4	24.8	20.3
95% CI^b^	(6.3, 45.0)	(5.4, 41.5)	(6.2, 44.5)	(4.7, 43.6)	(5.9, 43.5)	(5.3, 44.9)	(7.9, 46.9)	(9.0, 47.8)	(4.8, 43.6)

a Posterior median.

b 95% credible interval.

### 3.3 Sensitivity to prior assumptions

The choice of prior distributions (i.e., non‐informative vs informative) has some influence on the fit of the model, with less informative combinations generally resulting in a slightly better fit to the data (Fig. S1). In addition, when a non‐informative prior is used for the natural mortality rate, the baseline level of mortality is typically higher than when an informative one is used (Fig. S1).

Estimates for all parameters show sensitivity to prior assumptions, with some general trends for each parameter, but ones which do not necessarily apply to all herds (Fig. S2). The basic *R*_0_ is typically higher for any combination of priors which include at least one non‐informative prior. Transmission rates are often, though not always, much higher when a non‐informative compared with an informative prior is used for that parameter. Posterior estimates for the mean latent period are typically higher when a non‐informative prior is used. By contrast, posterior estimates for the mean infectious period are often lower if a non‐informative prior is used. If a non‐informative prior is used, the shape parameters for either the latent or the infectious periods are much higher. Finally, the posterior estimates for the natural mortality rate when a non‐informative prior is used can be markedly higher than if an informative prior is used. In these cases, more of the observed mortality is ascribed to natural background mortality rather than to ASFV‐related mortality (compare, e.g., Fig. S1).

## 4 DISCUSSION

Despite ASF being one of the most important transboundary diseases of swine, relatively few studies have estimated transmission parameters for this disease (Guinat, Gogin, et al., 2016). In those studies which have estimated parameters, data from both transmission experiments (de Carvalho Ferreira, Backer, et al., 2013; Guinat, Gubbins, et al., 2016; Hu et al., 2017; Nielsen, Karsen, Halasa, & Christiansen, 2017; Pietschamnn et al., 2015) and field data (Gulenkin et al., 2011; Korennoy, Gulenkin, Gogin, Vergne, & Karaulov, 2017) have been used. This relative paucity of data is reflected in the ASFV modelling literature, where the models (see, e.g., Barongo, Bishop, Fèvre, Knobel, & Ssematimba, 2016; Halasa, Boklund, Bøtner, Toft, & Thulke, 2016; Mur et al., 2017) typically rely on parameters derived from three studies (Gulenkin et al., 2011; de Carvalho Ferreira, Backer, et al., 2013; de Carvalho Ferreira, Weesendorp, Quak, Stegeman, & Loeffen, 2013; Guinat, Gubbins, et al., 2016).

Our estimates for *R*_0_ of outbreaks in nine pig herds ranged from 4.4 to 17.3 (Table 1). Similar values have been reported previously for the strains of ASFV currently circulating in the RF, whether from outbreaks (9.8 in Gulenkin et al., 2011) or transmission experiments (5.3 in Guinat, Gubbins, et al., 2016; 24.1 in Hu et al., 2017). In addition, similar values have been reported for other ASFV strains: 7.5 for Ukraine 1977 (Korennoy et al., 2017); 18.0 for Malta 1978 (de Carvalho Ferreira, Backer, et al., 2013); and 4.9 for Netherlands 1986 (de Carvalho Ferreira, Backer, et al., 2013). Our estimates for transmission rates (Table 1) are also similar to those reported for the Georgia 2007 (Guinat, Gubbins, et al., 2016; Hu et al., 2017; Nielsen et al., 2017) and other (de Carvalho Ferreira, Backer, et al., 2013; Korennoy et al., 2017) strains.

Previous studies of ASFV have either assumed durations for the latent and infectious periods (Guinat, Gubbins, et al., 2016; Gulenkin et al., 2011; Korennoy et al., 2017) or inferred them using virus isolation data from transmission experiments (de Carvalho Ferreira, Backer, et al., 2013; Hu et al., 2017). Our estimates (Table 1) are the first ones based on field data from ASFV outbreaks. These suggest that the mean latent period is longer by 1 or 2 days (Figure 2c), and the mean infectious period is shorter by 1–4 days (Figure 2e) than was inferred from transmission experiments (Hu et al., 2017).

Most of the parameters differed amongst herds (Table 1; Figure 2). Differences in transmission rates will reflect the impact of behaviours of stockmen or others who may be able to introduce virus or influence the risk of transmission, as well as the effect of herd management practices on spread (Evans, Medley, Creasey, & Green, 2010; Lurette et al., 2008). However, the small number of herds in the study means that it is not feasible to identify particular behaviours or practices associated with higher (or lower) transmission rates (cf. Bos et al., 2009). In addition to transmission rates, the mean latent and infectious periods also differed amongst herds (Table 1; Figure 2). Such differences are unlikely to reflect breed or other genetic differences amongst pigs in the outbreak herds, as all breeds (and wild boar) are similarly susceptible to virulent ASFV isolates. Low doses of ASFV have been reported to result in prolonged incubation periods (Howey, O'Donnell, de Carvalho Ferreira, Borca, & Arzt, 2013; Pietschamnn et al., 2015) and, consequently, farm management practices or behaviours which influence the dose to which pigs are exposed could help account for the differences amongst herds.

Inferences about the transmission parameters drawn from the mortality data are sensitive to prior assumptions (Fig. S2). The informative priors used in this study (Table S3) represent the best information currently available for the strains of ASFV currently circulating in the RF (and more widely in eastern Europe). Furthermore, even though the priors are informative, they do still include substantial uncertainty (Figure 2).

We have ignored the potential impact of herd structure on transmission in the model. This is principally a consequence of the limited availability of data on how pigs were managed within the farms included in the study. Transmission experiments have shown that spread of ASFV between adjoining pens does occur and at rates sufficient to sustain an outbreak (i.e., the between‐pen *R*_0_^ ^> 1), though the rates are lower than within a pen (Guinat, Gubbins, et al., 2016; Hu et al., 2017). In addition to transmission through direct and indirect contact between adjoining pens, there would be other means of transmission between groups of pigs some distance apart, for example via fomites (Costard et al., 2013) or airborne spread (de Carvalho Ferreira, Weesendorp, et al., 2013). Estimating the transmission rate for each route using only mortality data would be challenging (any decrease in one route could be compensated for by an increase in another). Consequently, it is difficult to assess what impact neglecting herd structure would have on the estimates of transmission rates.

Our results have important implications for the control of ASFV. Given that there is currently no vaccine available against ASFV, control of ASF in countries in which housed commercial pig production systems predominate relies on biosecurity, movement restrictions and rapid detection and stamping out of affected herds (Costard et al., 2009; Guinat, Vergne, et al., 2016). In a recent survey of ASF experts, one of the preferred surveillance options for ASFV was syndromic surveillance of pig mortality (Guinat, Vergne, et al., 2016). Yet, our results suggest that ASFV could be circulating in a herd for nearly a month before it causes a marked increase in mortality (Figure 2h). This raises the question of how effective monitoring pig mortality would be as a method of early detection of ASFV, particularly during the earlier stages of an epidemic when farmer awareness may be limited. Such a long delay in detection could result in substantial within‐herd transmission (Figure 1), and mean a herd could pose a transmission risk to neighbouring herds for a substantial period of time (Porphyre et al., 2017). Inferences about the time of introduction are useful, however, as they help inform backward tracing of contacts between infected and susceptible herds (Elbers et al., 2001) and help improve predictions of epidemic characteristics (Porphyre et al., 2016).

An objective of the present study was to explore the feasibility of using mortality data to estimate transmission parameters, while making as few assumptions as possible. Our results show that this can be done: the posterior distributions for most model parameters differ from their prior distributions (Figure 2 and Fig. S2), indicating that there is information about them in the mortality data. Moreover, it is possible to draw inferences even using non‐informative priors, although any inferences are likely to be more reliable if there are data available to construct informative priors. Accordingly, the methods developed in this study could be applied to other high mortality diseases, notably highly pathogenic avian influenza. It would also be of interest to explore what level of mortality a disease must cause for mortality data to be useful when inferring transmission parameters and, hence, to which other diseases our approach could be applied.

## CONFLICT OF INTEREST

The authors declare no competing financial interests.
