# Supplementary material for: Inferring within‐herd transmission parameters for African swine fever virus using mortality data from outbreaks in the Russian Federation
Source: Transbound Emerg Dis. 2017 Nov 9;65(2):e264–71. doi: 10.1111/tbed.12748 (PMC5887875; doi:10.1111/tbed.12748)
Supplement: Supplementary file 1 [file TBED-65-e264-s001.docx]

**Text S1. Implementation of the approximate Bayesian computation sequential Monte Carlo scheme.**

Here we provide details of the implementation of the approximate Bayesian computation (ABC) sequential Monte Carlo (SMC) scheme used to estimate parameters in the ASFV model.

The ABC-SMC scheme is implemented as the following sequence of steps (McKinley et al. 2009; Toni et al. 2009):

1. Start at round *t*=1. Initialise the tolerance for the goodness-of-fit metric, *ε*_1_.
2. Generate a particle (i.e. set of parameters), *θ_i_*_,_*_t_*:
   1. if *t*=1, sample from the joint prior density, *π*(*θ*);
   2. if *t*>1, sample the particles with the weights *w_j_*_,_*_t_*_-1_ generated during the previous round; perturb the particle by adding a Uniform(-0.1ξ*_t_*_-1_,0.1ξ*_t_*_-1_) variate to each parameter, where ξ*_t_*_-1_ is the range of the marginal distribution for the parameter in the previous round (Conlan et al. 2012).
3. Run the model with the particle and calculate the simulated daily mortality.
4. Compare the simulated and observed daily mortalities using the goodness-of-fit metric, *D* (defined by equation (3) in the main paper). If *D*<*ε_t_*, accept the particle; otherwise (i.e. if *D*≥*ε_t_*), go back to step (2).
5. Calculate the weight for the particle,
6. if *t*=1, *w_i_*_,_*_t_*=1;
7. if *t*>1, the weight is given by,

$$w_{i,t}=\frac{\pi(\theta_{i,t})}{\sum_{j=1}^{N} w_{j,t-1}K(\theta_{i,t}|\theta_{j,t-1})},$$

where *π*(*θ_i,t_*) is the prior probability of the particle, *w_j_*_,_*_t_*_-1_ is the weight of particle *j* in the previous round and *K*(*θ_i,t_*|*θ_j,t_*_-1_) is the probability of moving from particle *j* to particle *i* (i.e. the perturbation kernel; see step (2b)).

1. Repeat steps (2)-(5) until 10,000 particles have been accepted.
2. Normalise the particle weights, so that they sum to one.
3. Calculate a new tolerance from the median value of the *D*s for the round (Conlan et al. 2012).
4. Increase the round number to *t*+1 and go to step (2).

Convergence of the posterior distributions was monitored by visual inspection of the outputs (posterior distributions and observed vs simulated mortality) from consecutive SMC rounds.

The acceptance tolerance (*ε_t_*) was set to 50 times the cumulative mortality for the herd on the first round. For each set of prior distributions (see main paper), convergence to the posterior distribution was achieved after 13-19 SMC rounds, with a final tolerance of around 0.5-1.5 times the cumulative mortality for the herd.

**References for Text S1**

Conlan, A.J.K., McKinley, T.J., Karolemeas, K., Brooks Pollock, E., Goodchild, A.V., Mitchell, A.P., Birch, C.P.D., Clifton-Hadley, R.S. & Wood, J.L.N. 2012 Estimating the hidden burden of bovine tuberculosis in Great Britain. *PLoS Comp. Biol.* **8**, e1002730.

McKinley, T., Cook, A.R. & Deardon, R. 2009 Inference in epidemic models without likelihoods. *Int. J. Biostat.* **5**, 24.

Toni, T., Welch, D., Strelowa, N., Ipsen, A. & Stumpf, M.P.H. 2009 Approximate Bayesian computation scheme for parameter inference and model selection in dynamical systems. *J. R. Soc. Interface* **6**, 187-202.
