# Supplementary figures and images for: Inferring within‐herd transmission parameters for African swine fever virus using mortality data from outbreaks in the Russian Federation
Source: Transbound Emerg Dis. 2017 Nov 9;65(2):e264–71. doi: 10.1111/tbed.12748 (PMC5887875; doi:10.1111/tbed.12748)

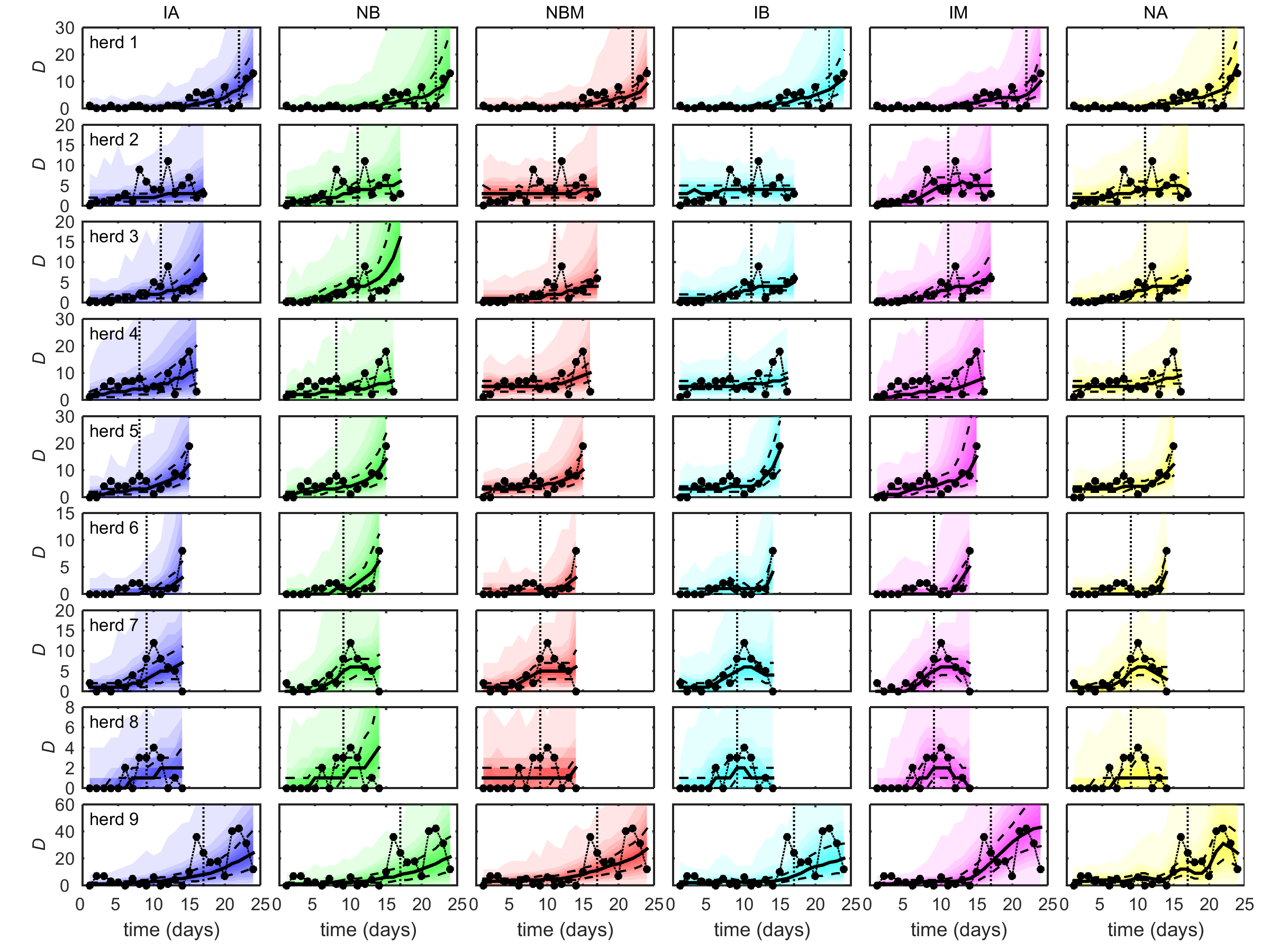

Supplement: Supplementary file 2 [file TBED-65-e264-s002.tiff]

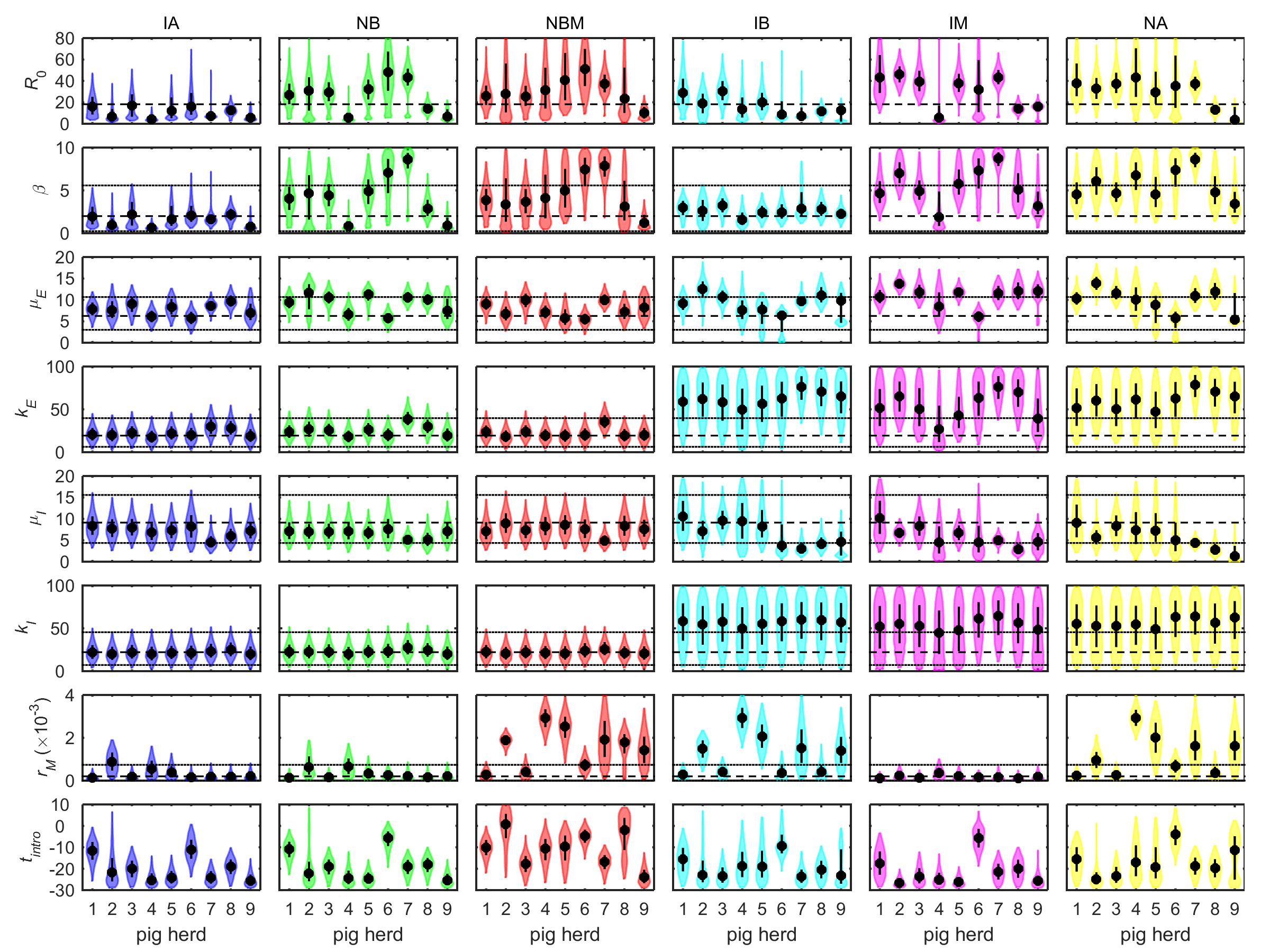

Supplement: Supplementary file 3 [file TBED-65-e264-s003.tiff]
