# Supplementary material for: Inferring within‐herd transmission parameters for African swine fever virus using mortality data from outbreaks in the Russian Federation
Source: Transbound Emerg Dis. 2017 Nov 9;65(2):e264–71. doi: 10.1111/tbed.12748 (PMC5887875; doi:10.1111/tbed.12748)
Supplement: Supplementary file 4 [file TBED-65-e264-s004.docx]

**Table S1.** Daily mortality data for nine pig herds in the Russian Federation infected with African swine fever virus.

| Observation period (day) | Herd number | | | | | | | | |
| --- | --- | --- | --- | --- | --- | --- | --- | --- | --- |
|  | 1 | 2 | 3 | 4 | 5 | 6 | 7 | 8 | 9 |
| 1 | 1 | 0 | 0 | 1 | 0 | 0 | 2 | 0 | 0 |
| 2 | 0 | 1 | 0 | 2 | 0 | 0 | 0 | 0 | 7 |
| 3 | 0 | 1 | 0 | 5 | 4 | 0 | 1 | 0 | 7 |
| 4 | 1 | 1 | 0 | 7 | 6 | 0 | 0 | 0 | 3 |
| 5 | 0 | 2 | 1 | 5 | 4 | 1 | 2 | 0 | 2 |
| 6 | 0 | 3 | 1 | 7 | 4 | 1 | 1 | 2 | 0 |
| 7 | 1 | 1 | 1 | 7 | 6 | 2 | 4 | 0 | 5 |
| 8 | 1 | 9 | 2 | 8* | 8* | 2 | 2 | 3 | 1 |
| 9 | 0 | 6 | 2 | 4 | 6 | 1* | 8* | 3* | 0 |
| 10 | 0 | 4 | 5 | 5 | 1 | 0 | 12 | 4 | 6 |
| 11 | 0 | 4* | 4* | 4 | 3 | 0 | 8 | 3 | 0 |
| 12 | 1 | 11 | 9 | 10 | 5 | 1 | 6 | 0 | 1 |
| 13 | 1 | 3 | 1 | 2 | 9 | 1 | 5 | 1 | 4 |
| 14 | 0 | 5 | 3 | 14 | 8 | 8 | 0 | 0 | 0 |
| 15 | 4 | 7 | 3 | 18 | 19 | † | † | † | 10 |
| 16 | 6 | 2 | 5 | 3 | † | - | - | - | 36 |
| 17 | 5 | 3 | 6 | † | - | - | - | - | 24* |
| 18 | 6 | † | † | - | - | - | - | - | 17 |
| 19 | 1 | - | - | - | - | - | - | - | 18 |
| 20 | 8 | - | - | - | - | - | - | - | 7 |
| 21 | 0 | - | - | - | - | - | - | - | 40 |
| 22 | 1* | - | - | - | - | - | - | - | 42 |
| 23 | 11* | - | - | - | - | - | - | - | 31 |
| 24 | 13 | - | - | - | - | - | - | - | 12 |
| 25 | † | - | - | - | - | - | - | - | † |
| Total number of dead pigs | 61 | 63 | 43 | 102 | 83 | 17 | 51 | 16 | 273 |
| Initial number of pigs | 1614 | 1949 | 1753 | 1833 | 1320 | 600 | 600 | 600 | 2145 |

*: day of ASFV-positive diagnosis

†: day of culling of all animals as part of disease control
