# Supplementary material for: Inferring within‐herd transmission parameters for African swine fever virus using mortality data from outbreaks in the Russian Federation
Source: Transbound Emerg Dis. 2017 Nov 9;65(2):e264–71. doi: 10.1111/tbed.12748 (PMC5887875; doi:10.1111/tbed.12748)
Supplement: Supplementary file 5 [file TBED-65-e264-s005.docx]

**Table S2.** Transitions, probabilities and population sizes in the model for the transmission of African swine fever virus within a farm.

| description | transition | probability | population size |
| --- | --- | --- | --- |
| infection | $\left\{\begin{matrix} S\to S-1 \\ E_{1}\to E_{1}+1 \end{matrix}\right.$ | *λδt* | *S* |
| completion of latent period stage *j*  (*j*=1,…,*k_E_*-1) | $\left\{\begin{matrix} E_{j}\to E_{j}-1 \\ E_{j+1}\to E_{j+1}+1 \end{matrix}\right.$ | (*k_E_*/*μ_E_*)*δt* | *E_j_* |
| completion of latent period | $\left\{\begin{matrix} E_{k_{E}}\to E_{k_{E}}-1 \\ I_{1}\to I_{1}+1 \end{matrix}\right.$ | (*k_E_*/*μ_E_*)*δt* | *E_j_* |
| natural mortality during latent period stage *j*  (*j*=1,…,*k_E_*) | $E_{j}\to E_{j}-1$ | *r_M_δt* | *E_j_* |
| completion of infectious period stage *j*  (*j*=1,…,*k_I_*-1) | $\left\{\begin{matrix} I_{j}\to I_{j}-1 \\ I_{j+1}\to I_{j+1}+1 \end{matrix}\right.$ | (*k_I_*/*μ_I_*)*δt* | *I_j_* |
| disease-associated mortality at end of infectious period | $I_{k_{I}}\to I_{k_{I}}-1$ | (*k_I_*/*μ_I_*)*δt* | *I_j_* |
| natural mortality during infectious period stage *j*  (*j*=1,…,*k_I_*) | $I_{k_{I}}\to I_{k_{I}}-1$ | *r_M_δt* | *I_j_* |
