# Supplementary material for: Inferring within‐herd transmission parameters for African swine fever virus using mortality data from outbreaks in the Russian Federation
Source: Transbound Emerg Dis. 2017 Nov 9;65(2):e264–71. doi: 10.1111/tbed.12748 (PMC5887875; doi:10.1111/tbed.12748)
Supplement: Supplementary file 6 [file TBED-65-e264-s006.docx]

**Table S3.** Prior distributions used for parameters in the model for the within-herd transmission of African swine fever virus.

| parameter | symbol | prior distribution | |
| --- | --- | --- | --- |
|  |  | informative | non-informative |
| within-herd transmission rate | *β* | Gamma(2,2) | Uniform(0,10) |
| mean duration of latent period | *µ_E_* | Gamma(6.25,10) | Uniform(0,20) |
| shape parameter for latent period | *k_E_* | Gamma(19.39,5) | Uniform(0,100) |
| mean duration of infectious period | *µ_I_* | Gamma(9.12,10) | Uniform(0,20) |
| shape parameter for infectious period | *k_I_* | Gamma(22.20,5) | Uniform(0,100) |
| natural mortality rate | *r_M_* | Exponential(0.0002) | Uniform(0,0.005) |
